# Supplementary material for: Microbiological Etiology in Patients with IE Undergoing Surgery and for Patients with Medical Treatment Only: A Nationwide Study from 2010 to 2020
Source: Microorganisms. 2023 Sep 26;11(10):2403. doi: 10.3390/microorganisms11102403 (PMC10608926; doi:10.3390/microorganisms11102403)

Supplemental Material

Table of content

Table S1: Overview of codes used to define study population, co-medication, and comorbidity.................... 3

Table S2a: *Streptococcus* species - distribution on species level.. 5

Table S2b: Coagulase-negative Staphylococci - distribution on species level.. 6

Table S2c: Other microbiological causes - distribution on species level 7

Table S3: Frequencies and proportions of the heart valves replaced in the surgical treated patients............... 8

Table S4: Baseline characteristics in the age -and sex-matched cohort stratified by treatment choice............ 9

Table S5: Baseline characteristics stratified by microbiological etiology in patients who underwent surgery. 11

Table S6: Overview of status at discharge (alive or dead) in numbers and percentages according to microbiological etiology and treatment choice 13

Figure S1: Proportion of treatment choice stratified by microbiological etiologies after exclusion of CIED at baseline.. 14

Figure S2: Proportion of microbiological etiologies stratified by treatment choice (surgery during admission vs. medical therapy) after exclusion of CIED at baseline 15

Figure S3: Proportion of microbiological etiologies stratified by treatment choice (surgery during admission vs. medical therapy) in an age – and sex-matched cohort.. 16

Figure S4: Cumulative incidence of surgery during admission stratified by microbiological etiology......... 17

Figure S5A+B: Mortality rates by microbiological etiology and treatment choice (surgery during admission vs. medical therapy) in an age -and sex-matched cohort.. 18

Table S1: Overview of codes used to define study population, co-medication, and comorbidity

Category	Codes
Study population	
Infective endocarditis (first-time event)	ICD-10: DI33, DI38, DI398; ICD8: 421
Surgery during admission	NOMESCO
Aortic valve	Bioprostheses: KFMD10, KFCA70<br>Mechanical prostheses: KFMD00, KFCA60<br>Other valve prostheses: KFMD20, KFMD30, KFMD33, KFMD96
Mitral valve	KFK
Pulmonic valve	KFJB, KFJC, KFJE, KFJF, KFJW
Tricuspid valve	KFG
Comorbidity/previous intervention (prior to index date)	
Aortic valve disease	ICD-10: I35s<br>ICD-8: 395, 396
Atrial flutter/fibrillation	ICD-10: DI48,<br>ICD-8:42793, 42794
Chronic obstructive lung disease (COPD)	ICD-10: DJ42-44<br>ICD-8: 490-492
Chronic kidney disease (CKD)	ICD-10: DN02-08, DN11-12, DN14, DN18-19, DN26, DN158-160, DN162-164, DN168, DQ612-613, DQ615, DQ619, DE102, DE112, DE132, DE142, DI120, DI131, DM300, DM313, DM319, DM321, DZ992<br>ICD-8: 403-404, 581-584, 25002, 40039, 59009, 59320, 75310, 754311, 75319
CIED	NOMESCO: BFCA0, BFCB0
Diabetes mellitus	ICD-10: DE10-14<br>ICD-8: 250<br>ATC code: A10
Heart failure	ICD10: DI42, DI50, DI099, DI110, DI130, DI132, DJ819<br>ICD-8: 425, 428, 4270, 4271, 78249
Ischemic heart disease (IHD)	ICD-10: DI20-25<br>ICD-8: 410-414
PCI	NOMESCO: KFNG0, KFNG10, KFNG12, KFNG96
CABG	NOMESCO: KFNA, KFNB, KFNC, KFNC, KFNE
Liver disease	ICD-10: DB15-19, DK70-77, DC22, DI982, DZ944, DD684C, DQ618A,<br>ICD-8: 571-573, 155, 070
Malignancy	ICD-10: DC00-97, not including C44 (skin cancer)<br>ICD-8: 140-209, not including 173 (skin cancer)
Mitral valve disease	ICD-10: I34

	ICD-8: 394, 396
Prosthetic heart valve	KFKD, KFMD, KFGE, KFJF, KFCA60, KFCA70
Renal dialysis	ICD-10: DZ992<br>NOMESCO: BFJD2
Pharmacotherapy (within 6 months prior to index date)	
Anticoagulants (DOAC and VKA)	ATC code: B01AA, B01AE, B01AF
Beta blockers	ATC code: C07, C09BX
Lipid-lowering medication	ATC code: C10
RAS-inhibitors	ATC code: C09
Hypertension	*Defined as two or more antihypertensive drugs within 6 months prior to index data including the following:*
Adrenergic antagonists	ATC code: C02A, C02B, C02C
Beta blockers	ATC code: C07, C09BX
Calcium channel blockers	ATC code: C08, C07F, C09BB, C09DB
Combined diuretics	ATC code: C07C, C08G, C03B, C09BA, C09DA
Loop-diuretics	ATC code: C03C, C03EB01, C03EB02
MRA	ATC code: C03DA01-C03DA04
RAS-inhibitors	ATC code: C09
Thiazide diuretics	ATC code: C03A, C07B, C07D, C09XA52, C03EA01
Vasodilating drugs	ATC code: C02DB, C02DD, C02DG

ICD: international classification of diseases, ATC: Anatomical Therapeutical Classification System, AMI: acute myocardial infarction, PCI: percutaneous coronary intervention, CABG: coronary artery bypass grafting, CIED: cardiac implantable electronic device, DOAC: direct oral anticoagulant therapy, MRA: aldosterone receptor antagonists, RAS: renin angiotensin system, VKA: vitamin K antagonists

Table S2a: *Streptococcus* species - distribution on species level

Microorganism	Counts	Percent
**Non-hemolytic streptocci**		
S. anginosus / S. milleri group		
S. anginosus	67	2.82
S. milleri	13	0.74
S. constellatus	4	0.23
S. intermedius	6	0.34
S. bovis / equinus group		
S. bovis	163	9.30
S. gallolyticus	39	2.23
S. infantarius	7	0.40
S. mitis group		
S. mitis	385	21.97
S. gordonii	36	2.05
S. oralis	33	1.88
S. parasanguinis	10	0.57
S. sanguinis	108	6.16
S. pneumoniae	77	4.39
S. salivarius	55	3.14
S. mutans	98	5.59
**Non-hemolytic streptocci unspecified**	116	6.62
**Hemolytic streptococci**		
Hemolytic streptococci unspecified	43	245
Hemolytic streptococci group A	32	1.83
Hemolytic streptococci group B	149	8.50
Hemolytic streptococci group C	60	3.42
Hemolytic streptococci group G	133	7.59
Streptococcus dysgalactiae	33	1.88
**Other**		
Abiotrophia defectiva	23	1.31
Gemella haemolysans	5	0.29
Granulicatella adiacens	17	0.97
Granulicatella elegans	4	0.23
Streptococcus species unspecified	36	2.05

Table S2b: Coagulase-negative Staphylococci - distribution on species level

Microorganism	Counts	Percent
Bacteria anonymized due to count below 3	4	0.97
Staphylococcus capitis	28	6.76
Staphylococcus epidermidis	243	58.70
Staphylococcus haemolyticus	12	2.90
Staphylococcus hominis	25	6.04
Staphylococcus lugdunensis	43	10.39
Staphylococcus species unspecified	55	13.29
Staphylococcus warneri	4	0.97

Table S2c: Other microbiological causes - distribution on species level

Microorganism	Count	Percent
**HACAK**		
Haemophilus parainfluenzae	13	3.98
Aggregatibacter aphrophilus	8	2.45
Aggregatibacter actinomycetemcomitans	17	5.2
Cardiobacterium hominis	13	3.98
Cardiobacterium species	3	0.92
Kingella kingae	5	1.53
**Gram-positive bacteria**		
Aerococcus species	4	1.22
Aerococcus urinae	29	8.87
Corynebacterium striatum	4	1.22
Lacticaseibacillus rhamnosus	3	0.92
Lacticaseibacillus species	3	0.92
Listeria monocytogenes	6	1.83
Micrococcus species	3	0.92
Rothia dentocariosa	3	0.92
**Gram-negative bacteria**		
Enterobacter cloacae	8	2.45
Escherichia coli	40	12.23
Haemophilus influenzae	5	1.53
Klebsiella oxytoca	9	2.75
Klebsiella pneumoniae	15	4.59
Pseudomonas aeruginosa	12	3.67
Serratia marcescens	8	2.45
**Anaerobic**		
Bacteroides fragilis	3	0.92
Cutibacterium acnes	21	6.42
**Fungus**		
Candida albicans	8	2.45
Candida glabrata	5	1.53
**Unspecified**	13	3.98
**Bacteria anonymized due to count below 3**	66	20.18

Table S3: Frequencies and proportions of the heart valves replaced in the surgical treated patients

Surgical valve replacements	Numbers	Proportion (%)
**Left-sided valves**	**1184**	**92.79**
Isolated aortic valve[a]	632	53.38
Isolated mitral valve[a]	343	28.97
Combined (aortic and mitral valves)[a]	209	17.65
**Right-sided valves**	**31**	**2.43**
**Combined left -and right-sided valves**	**61**	**4.78**
Aortic valve and right-sided valves[b]	28	45.90
Mitral valve and right-sided valves[b]	13	21.31
Both aortic and mitral valves and right-sided valves[b]	20	32.79

[a]Frequencies and proportions of left-sided valve replacements. The percentages add up to 100% of all 1184 solely left-sided valve replacements.

[b]Frequencies and proportions of combined left -and right-sided valve replacements. The percentages add up to 100% of all 66 combined left -and right-sided valve replacements.

Table S4: Baseline characteristics in the age -and sex-matched cohort stratified by treatment choice

Variable	Medical therapy only, N = 1270[a]	Surgery during admission, N = 1270[a]
Males	973 (76.6)	973 (76.6)
Age	65 [54-72]	65 [54-72]
Length of hospital stay (days)	36 [26-48]	46 [35-58]
Length of hospital stay among patients alive at discharge (days)	41 [30-49]	46 [39-59]
Microbiological etiology		
Staphylococcus aureus	478 (37.6)	262 (20.6)
Streptococcus	310 (24.4)	444 (35.0)
Enterococcus	153 (12.0)	183 (14.4)
CoNS	93 (7.3)	91 (7.2)
Other	60 (4.7)	77 (6.1)
Negative	176 (13.9)	213 (16.8)
Prostethic heart valve	210 (16.5)	246 (19.4)
Cardiac implantable electrical devices (CIED)	235 (18.5)	70 (5.5)
Aortic valve disease	253 (19.9)	362 (28.5)
Mitral valve disease	82 (6.5)	124 (9.8)
Atrial fibrillation (AF)	253 (19.9)	189 (14.9)
Heart failure (HF)	231 (18.2)	155 (12.2)
Ischemic heart disease (IHD)	334 (26.3)	226 (18.8)
Cerebrovascular disease (CVD)	176 (13.9)	149 (11.7)
Dementia	14 (1.1)	≤3
Hypertension	622 (49.0)	512 (40.3)
Chronic kidney disease (CKD)	137 (10.8)	78 (6.1)
Dialysis	91 (7.2%)	53 (4.2)
Diabetes	309 (24.3)	202 (15.9)
Liver disease	114 (9.0)	35 (2.8)
Chronic obstructive pulmonary disease (COPD)	128 (10.1)	76 (6.0)
Charlson comorbidity index		

Table S4: Baseline characteristics in the age -and sex-matched cohort stratified by treatment choice

Variable	Medical therapy only, N = 1270[a]	Surgery during admission, N = 1270[a]
0	454 (35.7)	704 (55.4)
1-2	432 (34.0)	412 (32.4)
>2	384 (30.2)	154 (12.1)
Anticoagulants	322 (25.4)	260 (20.5)
Beta blockers	481 (37.9)	336 (26.5)
Lipid-lowering medication	462 (36.4)	409 (32.2)
RAS inhibitors	498 (39.2)	464 (36.5)

[a]Median [25th.75th percentile] or Frequency (%)

Table S5: Baseline characteristics stratified by microbiological etiology in patients who underwent surgery.

Variable	*S. aureus*, N = 262[a]	*Streptococcus* spp., N = 447[a]	*Enterococcus* spp., N = 183[a]	CoNS, N = 92[a]	Other, N = 77[a]	Negative, N = 215[a]
Males	180 (68.7%)	349 (78.1%)	153 (83.6%)	67 (72.8%)	65 (84.4%)	159 (74.0%)
Age	61.0 [49.0-70.0]	64.0 [55.0-71.5]	71.0 [64.0-76.0]	65.5 [57.0-73.0]	64.0 [53.0-71.0]	62.0 [51.0-71.5]
Length of hospital stay (days)	51.0 [43.0-67.0]	43.0 [32.0-51.0]	50.0 [44.0-65.0]	46.0 [42.0-58.2]	47.0 [34.0-60.0]	45.0 [31.5-54.0]
Prior valve prosthesis	46 (17.6%)	67 (15.0%)	39 (21.3%)	33 (35.9%)	13 (16.9%)	50 (23.3%)
Cardiac implantable electrical devices (CIED)	15 (5.7%)	5 (1.1%)	23 (12.6%)	7 (7.6%)	7 (9.1%)	13 (6.0%)
Aortic valve disease	52 (19.8%)	99 (22.1%)	70 (38.3%)	37 (40.2%)	26 (33.8%)	78 (36.3%)
Mitral valve disease	16 (6.1%)	35 (7.8%)	14 (7.7%)	20 (21.7%)	6 (7.8%)	33 (15.3%)
Atrial fibrillation (AF)	35 (13.4%)	56 (12.5%)	31 (16.9%)	26 (28.3%)	12 (15.6%)	29 (13.5%)
Heart failure (HF)	25 (9.5%)	33 (7.4%)	33 (18.0%)	24 (26.1%)	8 (10.4%)	33 (15.3%)
Ischemic heart disease (IHD)	38 (14.5%)	62 (13.9%)	50 (27.3%)	19 (20.7%)	15 (19.5%)	42 (19.5%)
Hypertension	109 (41.6%)	142 (31.8%)	96 (52.5%)	41 (44.6%)	27 (35.1%)	97 (45.1%)
Chronic kidney disease (CKD)	31 (11.8%)	12 (2.7%)	9 (4.9%)	12 (13.0%)	4 (5.2%)	10 (4.7%)
Dialysis	26 (9.9%)	3 (0.7%)	7 (3.8%)	11 (12.0%)	≤3 (<4.0%)	5 (2.3%)

Table S5: Baseline characteristics stratified by microbiological etiology in patients who underwent surgery.

Variable	*S. aureus*, N = 262[a]	*Streptococcus* spp., N = 447[a]	*Enterococcus* spp., N = 183[a]	CoNS, N = 92[a]	Other, N = 77[a]	Negative, N = 215[a]
Diabetes	46 (17.6%)	65 (14.5%)	33 (18.0%)	16 (17.4%)	13 (16.9%)	29 (13.5%)
Liver disease	10 (3.8%)	12 (2.7%)	3 (1.6%)	4 (4.3%)	3 (3.9%)	3 (1.4%)
Chronic obstructive pulmonary disease (COPD)	16 (6.1%)	19 (4.3%)	14 (7.7%)	6 (6.5%)	≤3 (<4.0%)	19 (8.8%)
Anticoagulants	47 (17.9%)	68 (15.2%)	55 (30.1%)	29 (31.5%)	20 (26.0%)	41 (19.1%)
Beta blockers	72 (27.5%)	87 (19.5%)	58 (31.7%)	36 (39.1%)	22 (28.6%)	61 (28.4%)
Lipid-lowering medication	80 (30.5%)	137 (30.6%)	78 (42.6%)	33 (35.9%)	22 (28.6%)	59 (27.4%)
RAS inhibitors	101 (38.5%)	141 (31.5%)	86 (47.0%)	35 (38.0%)	25 (32.5%)	76 (35.3%)

[a]Median [25th.75th percentile] or Frequency (%)

Table S6: Overview of status at discharge (alive or dead) in numbers and percentages according to microbiological etiology and treatment choice

	S. aureus (N=1924)	*Streptococcus* spp. (N=1752)	*Enteroccocus* spp. (N=982)	CoNS (N=414)	Other (N=327)	Negative (N=856)	Total (N=6255)
Surgery and death during admission	48 (2.5%)	50 (2.8%)	20 (2.0%)	9 (2.2%)	9 (2.8%)	19 (2.2%)	155 (2.5%) *(12.1%)**
Surgery and alive at discharge	214 (11.1%)	397 (22.7%)	163 (16.6%)	83 (20.1%)	68 (20.8%)	196 (22.9%)	1121 (17.9%) *(87.9%)**
Medical therapy and death during admission	489 (25.4%)	161 (9.2%)	147 (15.0%)	76 (18.4%)	40 (12.2%)	131 (12.6%)	1044 (16.7%) *(21.0%)***
Medical therapy and alive at discharge	1173 (61.0%)	1144 (65.3%)	652 (66.4%)	246 (59.4%)	210 (64.2%)	510 (59.6%)	3935 (62.9%) *(79.0%)***

**Out of all patients who underwent surgery during admission (N=1276)*
*** Out of all patients who received medical therapy only (N=4979)*

Figure S1: Proportion of treatment choice stratified by microbiological etiologies after exclusion of CIED at baseline

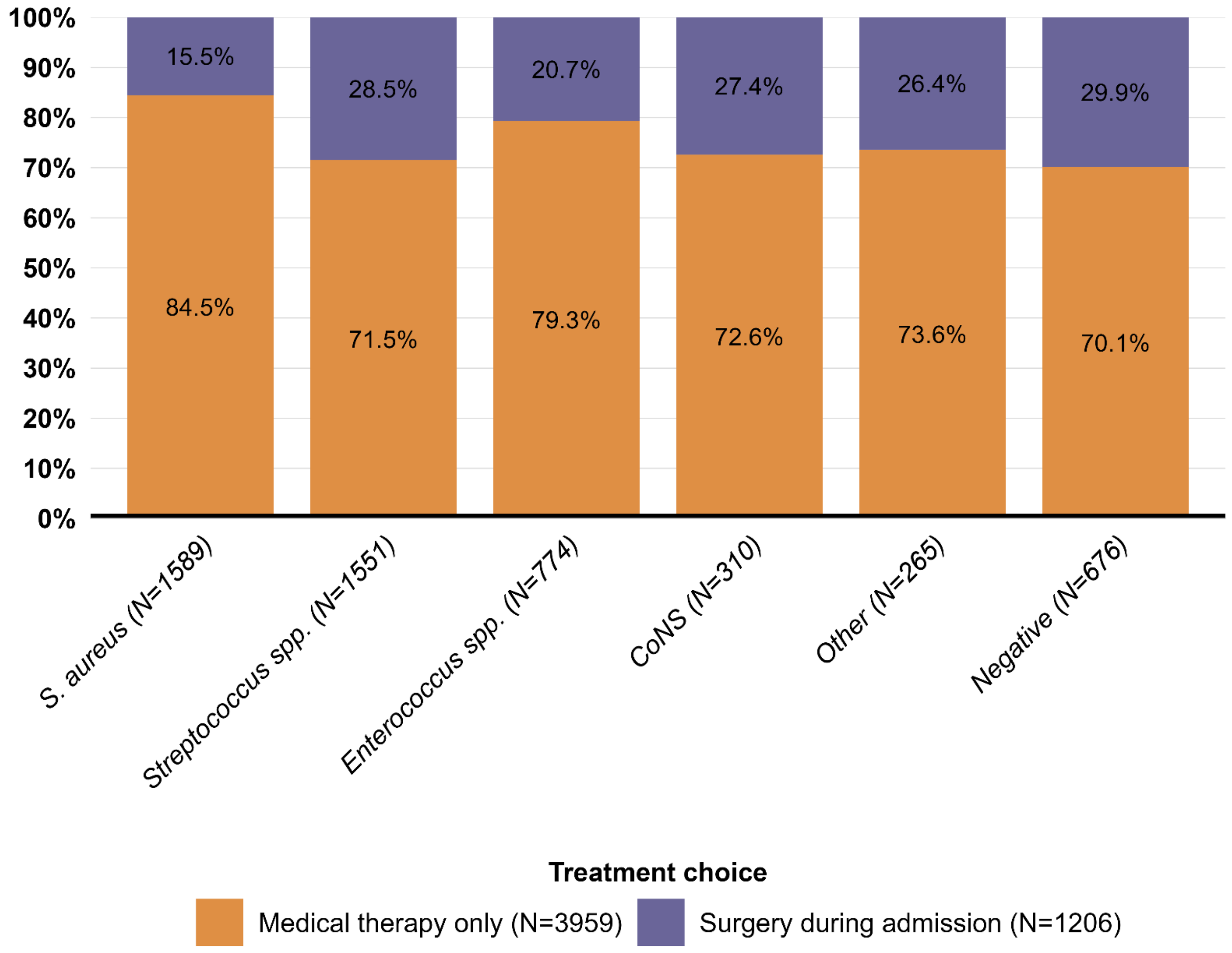

Figure S2: Proportion of microbiological etiologies stratified by treatment choice (surgery during admission vs. medical therapy) after exclusion of CIED at baseline

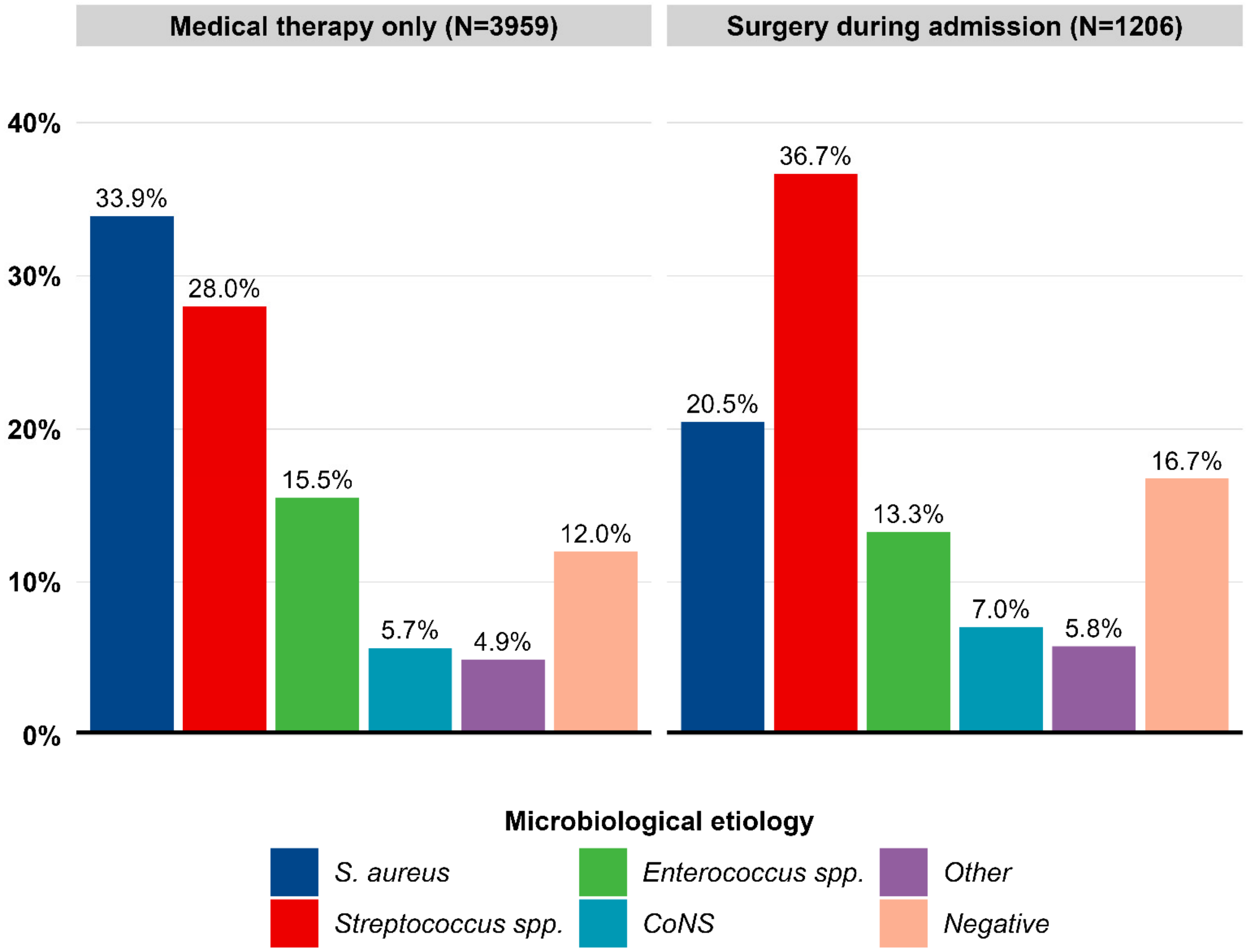

Figure S3: Proportion of microbiological etiologies stratified by treatment choice (surgery during admission vs. medical therapy) in an age – and sex-matched cohort

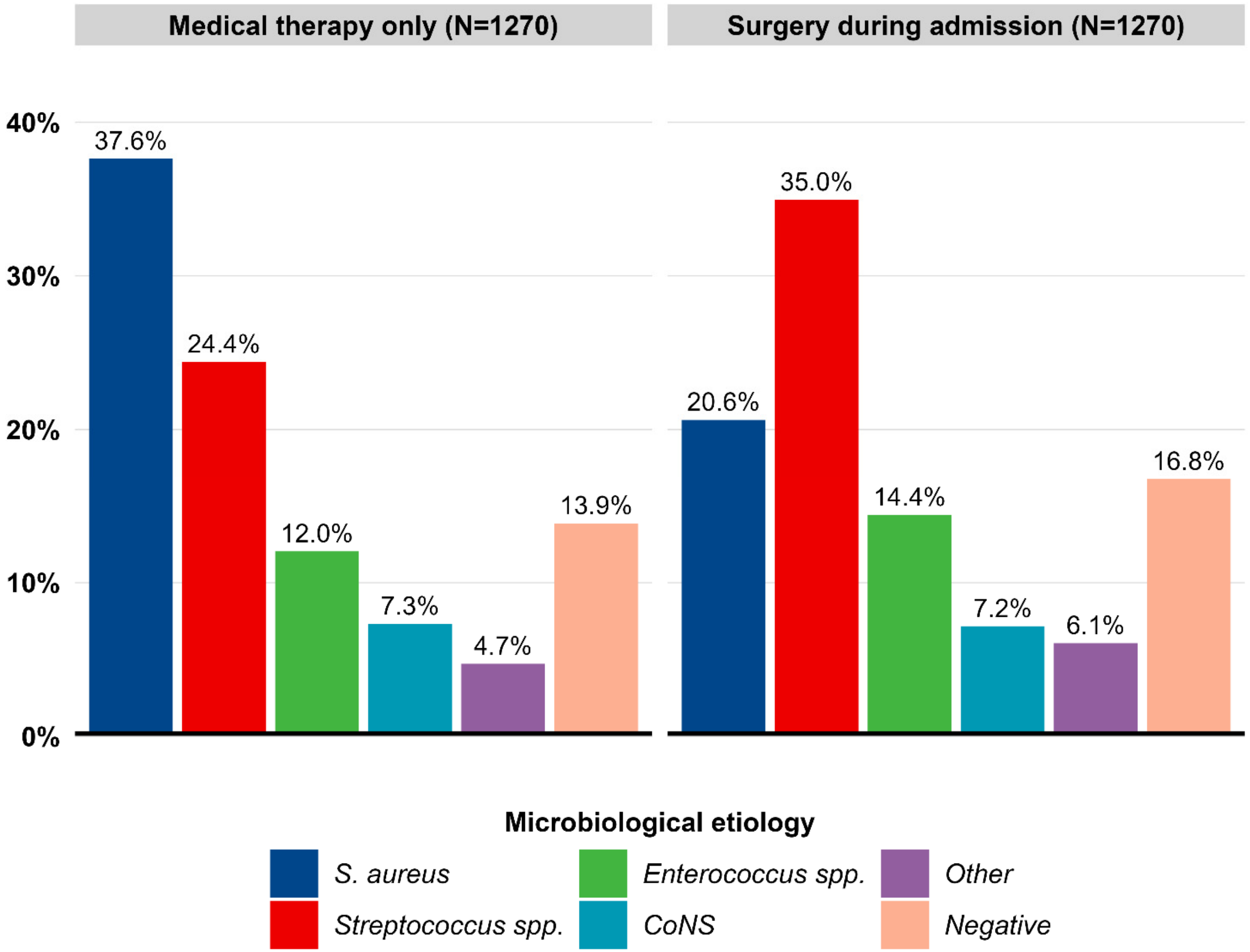

Figure S4: Cumulative incidence of surgery during admission stratified by microbiological etiology

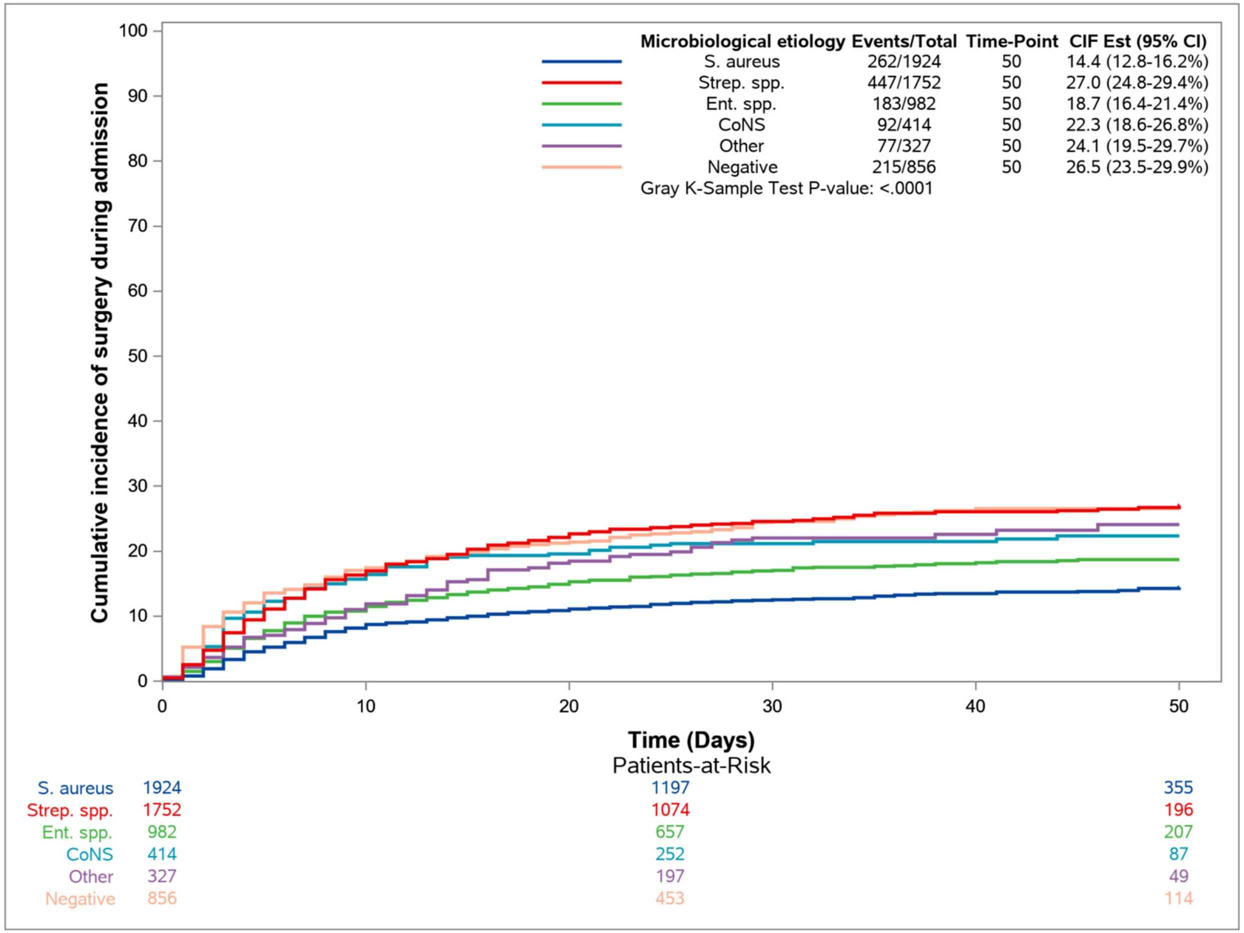

Figure S5A+B: Mortality rates by microbiological etiology and treatment choice (surgery during admission vs. medical therapy) in an age -and sex-matched cohort.

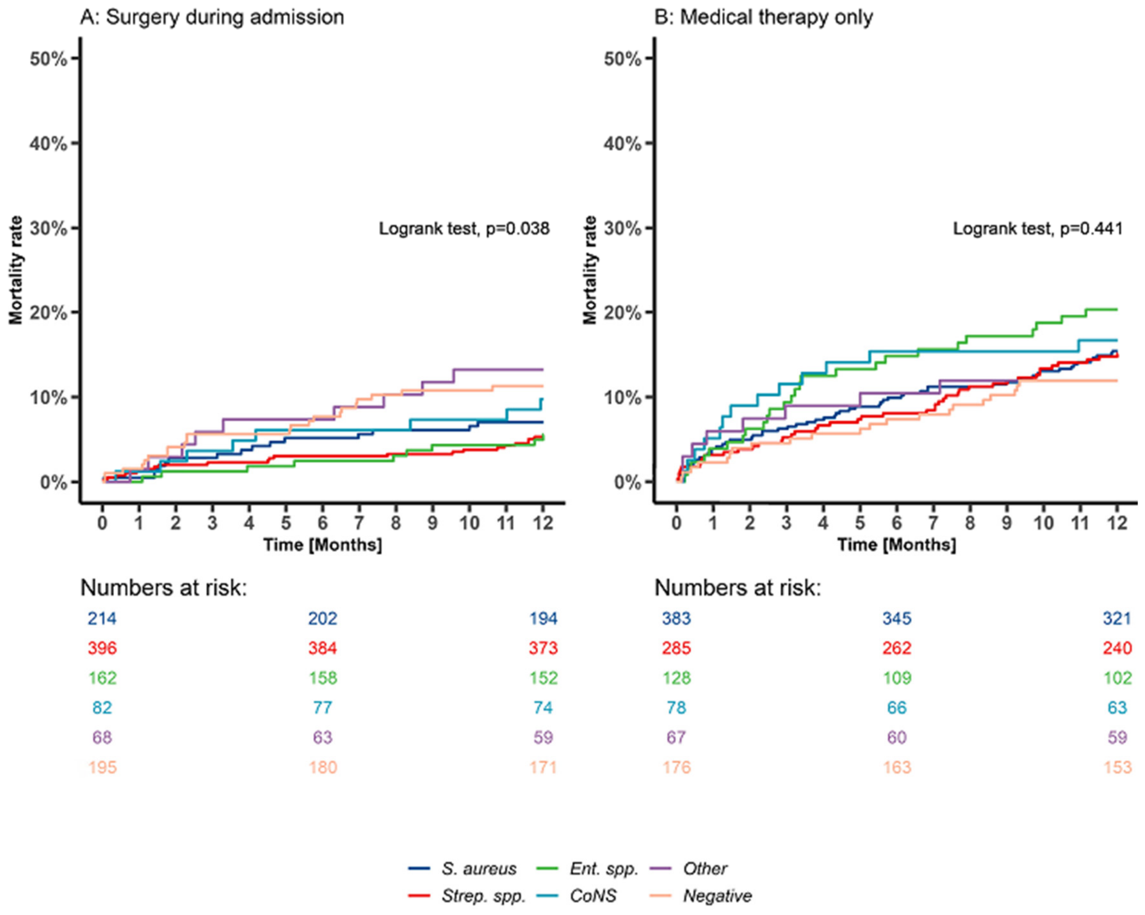

Supplement: Supplementary file 1 [file microorganisms-11-02403-s001.zip › microorganisms-2525823-supplementary.pdf]
